# Urate Crystal Induced Inflammation and Joint Pain Are Reduced in Transient Receptor Potential Ankyrin 1 Deficient Mice – Potential Role for Transient Receptor Potential Ankyrin 1 in Gout

**DOI:** 10.1371/journal.pone.0117770

**Published:** 2015-02-06

**Authors:** Lauri J. Moilanen, Mari Hämäläinen, Lauri Lehtimäki, Riina M. Nieminen, Eeva Moilanen

**Affiliations:** The Immunopharmacology Research Group, University of Tampere School of Medicine and Tampere University Hospital, Tampere, Finland; University of South California, UNITED STATES

## Abstract

**Introduction:**

In gout, monosodium urate (MSU) crystals deposit intra-articularly and cause painful arthritis. In the present study we tested the hypothesis that Transient Receptor Poten-tial Ankyrin 1 (TRPA1), an ion channel mediating nociceptive signals and neurogenic in-flammation, is involved in MSU crystal-induced responses in gout by utilizing three experi-mental murine models.

**Methods:**

The effects of selective pharmacological inhibition (by HC-030031) and genetic depletion of TRPA1 were studied in MSU crystal-induced inflammation and pain by using 1) spontaneous weight-bearing test to assess MSU crystal-induced joint pain, 2) subcutaneous air-pouch model resembling joint inflammation to measure MSU crystal-induced cytokine production and inflammatory cell accumulation, and 3) MSU crystal-induced paw edema to assess acute vascular inflammatory responses and swelling.

**Results:**

Intra-articularly injected MSU crystals provoked spontaneous weight shift off from the affected limb in wild type but not in TRPA1 knock-out mice referring alleviated joint pain in TRPA1 deficient animals. MSU crystal-induced inflammatory cell infiltration and accumulation of cytokines MCP-1, IL-6, IL-1beta, MPO, MIP-1alpha and MIP-2 into subcu-taneous air-pouch (resembling joint cavity) was attenuated in TRPA1 deficient mice and in mice treated with the selective TRPA1 inhibitor HC-030031 as compared to control animals. Further, HC-030031 treated and TRPA1 deficient mice developed tempered inflammatory edema when MSU crystals were injected into the paw.

**Conclusions:**

TRPA1 mediates MSU crystal-induced inflammation and pain in experimental models supporting the role of TRPA1 as a potential mediator and a drug target in gout flare.

## Introduction

Gout is an increasing inflammatory disease with a prevalence of 1–2%. Gout flares are characterized by acute burning arthritis with local hyperalgesia and pain caused by monosodium urate (MSU) crystals accumulated into the affected joint. [1] In inflammatory cells, non-soluble MSU crystals trigger the formation of reactive oxygen species (ROS) and activate inflammatory signaling cascades such as phosphoinositide 3-kinase (PI3K) and nuclear factor- κB (NF-κB) pathways, and NALP3 inflammasome. Activation of NALP3 results in caspase-1 mediated cleavage of pro-interleukin-1β to the functional inflammatory cytokine interleukin-1β (IL-1β). [2,3] IL-1β has been investigated as a potential therapeutic target in acute gouty arthritis and it has been found to mediate partly, but not fully, inflammatory and analgesic responses in MSU crystal-induced inflammation [4].

Transient Receptor Potential Ankyrin 1 (TRPA1) is a Ca^2+^ permeable ion channel involved in cold allodynia, nociception, and according to the recent findings, also in inflammation [5–10]. Since its discovery in 1999 [11] TRPA1 has drawn increasing interest as a therapeutic target to treat neuropathic and inflammatory pain [12,13]. TRPA1 was originally discovered in sensory neurons but later the expression and function of TRPA1 in various non-neuronal cells has been established [5]. TRPA1 is activated by noxious cold and a spectrum of naturally occurring irritating compounds, e.g. allyl isothiocyanate in mustard oil or allicin in garlic. Interestingly, many reactive molecules formed in inflammation, such as hydrogen peroxide, 4-hydroxynonenal, nitrooleic acid and some arachidonic acid metabolites also activate TRPA1 [5,14,15]. Furthermore, inflammatory signaling pathways, such as protein kinase A and phospholipase C, are known to sensitize TRPA1 [16]. In addition to the regulation of analgesic signals, activation of neuronal TRPA1 contributes to neurogenic inflammation by releasing proinflammatory neuropeptides calcitonin gene related peptide and substance P [5]. Together with the published data [17,18] our recent findings in a widely used animal model in anti-inflammatory drug research, i.e. carrageenan-induced paw inflammation [19], further suggest that TRPA1 is not only a target of exogenous noxious signals but also a significant endogenous mechanism involved in amplification of acute inflammation [19].

Pharmacological blockade and genetic depletion of TRPA1 have shown beneficial effects in several models of hyperalgesia and pain as well as in acute inflammation [5,6,8]. Therefore we hypothesized that TRPA1 may contribute to MSU crystal-induced inflammation and pain in gout flare. The aim of the present study was to address the hypothesis by investigating the effects of pharmacological inhibition and genetic depletion of TRPA1 in animal models evaluating MSU crystal-induced proinflammatory cytokine production, inflammatory edema and joint pain.

## Methods

### Animals

Wild type (WT) and TRPA1 knock-out (KO) B6;129P-Trpa1(tm1Kykw)/J mice (Charles River Laboratories, Sulzfeld, Germany) were used in the experiments. Mice were housed under standard conditions (12–12h light-dark cycle, 22±1°C) with food and water provided *ad libitum*. Animal experiments were carried out in accordance with the legislation for the protection of animals used for scientific purposes (Directive 2010/63/EU) and approved by The National Animal Experiment Board (approval number ESAVI/5250/04.10.03/2012, granted on September 3, 2012).

Intraperitoneal injection of medetomidine (0.5 mg/kg, Domitor, Orion Oyj, Espoo, Finland) and ketamine (75 mg/kg, Ketalar, Pfizer Oy Animal Health, Helsinki, Finland) were used for anesthesia. Animals were sacrificed after experiments by carbon monoxide followed by cranial dislocation. Reagents were purchased from Sigma Chemical Co., St. Louis, MO, USA unless otherwise indicated.

### Studied Animal Groups

To study drug effects, WT mice were dosed orally with the selective TRPA1 antagonist HC-030031 [20] (300 mg/kg), with the control compound dexamethasone (2 mg/kg) with known anti-inflammatory activity or with the vehicle 2h prior to the experiments. All drugs used were diluted in 75% polyethylene glycol and given by gastric gavage in a volume of 250 μl. The dose of HC-030031 was based on our previous studies and literature [19,21,22]. Effects of genetic depletion of TRPA1 were studied by comparing responses in TRPA1 KO and corresponding WT mice.

### Subcutaneous Air-Pouch Test

Subcutaneous air-pouch was created by injecting 3 ml (1^st^ day) and 1.5 ml (3^rd^ day) of sterile air into the dorsal skin of the studied mice under anaesthesia and after 7 days a synovial-like epithelium was present in the air-pouch [23]. The inflammation was induced by injecting 3 mg of MSU crystals prepared as described below diluted in 1 ml of sterile endotoxin free phosphate-buffered saline (PBS) into the air-pouch of the anesthetized mice. After 6h the mice were sacrificed and the exudate was harvested for cell-counting by hemocytometer and for cytokine measurements. Monocyte chemotactic protein-1 (MCP-1), interleukin-6 (IL-6), interleukin-1β (IL-1β), myeloperoxidase (MPO), macrophage inflammatory protein-1α (MIP-1α) and macrophage inflammatory protein-2 (MIP-2) were measured by enzyme-linked immunosorbent assay (ELISA) (R&D Systems Europe Ltd, Abindgon, UK).

### Paw Edema Test

Inflammatory paw edema was induced by injecting 0.5 mg of MSU crystals diluted in 40 μl of sterile endotoxin free PBS into the hind paw of anesthetized mice. Contralateral paw was injected with the corresponding volume of the vehicle and developed no measurable edema. The paw volume was measured up to 6h with plethysmometer (Ugo Basile, Comerio, Italy) and compared to the baseline value.

### Weight-Bearing Test

The MSU crystal-induced weight-bearing test originally described in 1986 [24,25] was triggered by injecting 0.5 mg of MSU crystals diluted in 40 μl of sterile endotoxin free PBS into the hind knee joint of anesthetized mice. Contralateral knee joint was injected with the corresponding volume of the vehicle. The willingness to bear weight on the affected joint was measured with an incapacitance meter (IITC Life Science, Woodland Hills, CA, USA) for four subsequent days and compared to the baseline value. The mice were habituated in the measurement room for 60 min prior to the measurement and the subsequent measurements were carried out at the same time of the day. To obtain reliable data on the weight distribution, each mouse was measured 8 times for 1 second at each time point.

### Preparation of MSU Crystals

MSU crystals were prepared as previously described [26] by diluting 1.0 g of uric acid into 200 ml of aqua adjusted to pH 14.0 with NaOH by heating and blending. Next, pH was lowered to 7.0 by adding HCl and MSU was crystallized overnight at room temperature in constant shaking. The formed crystals were filtered, washed, dried and re-suspended in PBS at a concentration of 50 mg/ml. In microscopic examination the MSU crystals were 5–20 μm in length. All used equipment and liquids were endotoxin free.

### Ca^2+^-Influx Measurement

TRPA1 mediated Ca^2+^-influx was measured in HEK293 cells [27] transiently transfected with plasmid encoding the human TRPA1 (pCMV6-XL4 from Origene, Rockville, MD, USA) as described previously [19]. Cultured cells were loaded with 4 μM fluo-3-acetoxymethyl ester and 0.08% Pluronic F-127 in Hanks’ balanced salt solution (HBSS) containing 1 mg/ml of bovine serum albumin, 2.5 mM probenecid and 25 mM HEPES (pH 7.2) for 30 minutes at room temperature. The intracellular free Ca^2+^ levels were assessed by Victor3 1420 multilabel counter (Perkin Elmer, Waltham, MA, USA) at excitation/emission wavelengths of 485/535 nm [28]. In the experiments, the cells were first pre-incubated with 100 μM of the TRPA1 antagonist HC-030031 [20] or the vehicle for 30 min at +37 C°. Thereafter, 1 mg/ml of MSU crystals or 50 μM AITC was added and the measurements were continued for 30s after which a robust Ca^2+^-influx was induced by application of 1 μM of control ionophore compound ionomycin. The concentrations of AITC and HC-030031 were based on our dose-response studies and literature [29,30].

### Statistical Analysis

Results are expressed as mean ± standard error of the mean (SEM). Data were analyzed with SPSS version 17.0 for Windows software (SPSS Inc, Chicago, IL, USA) by using Student’s *t*-test or one-way ANOVA with Bonferroni’s or Dunnett’s multiple comparison test.

## Results

**Fig 1:**
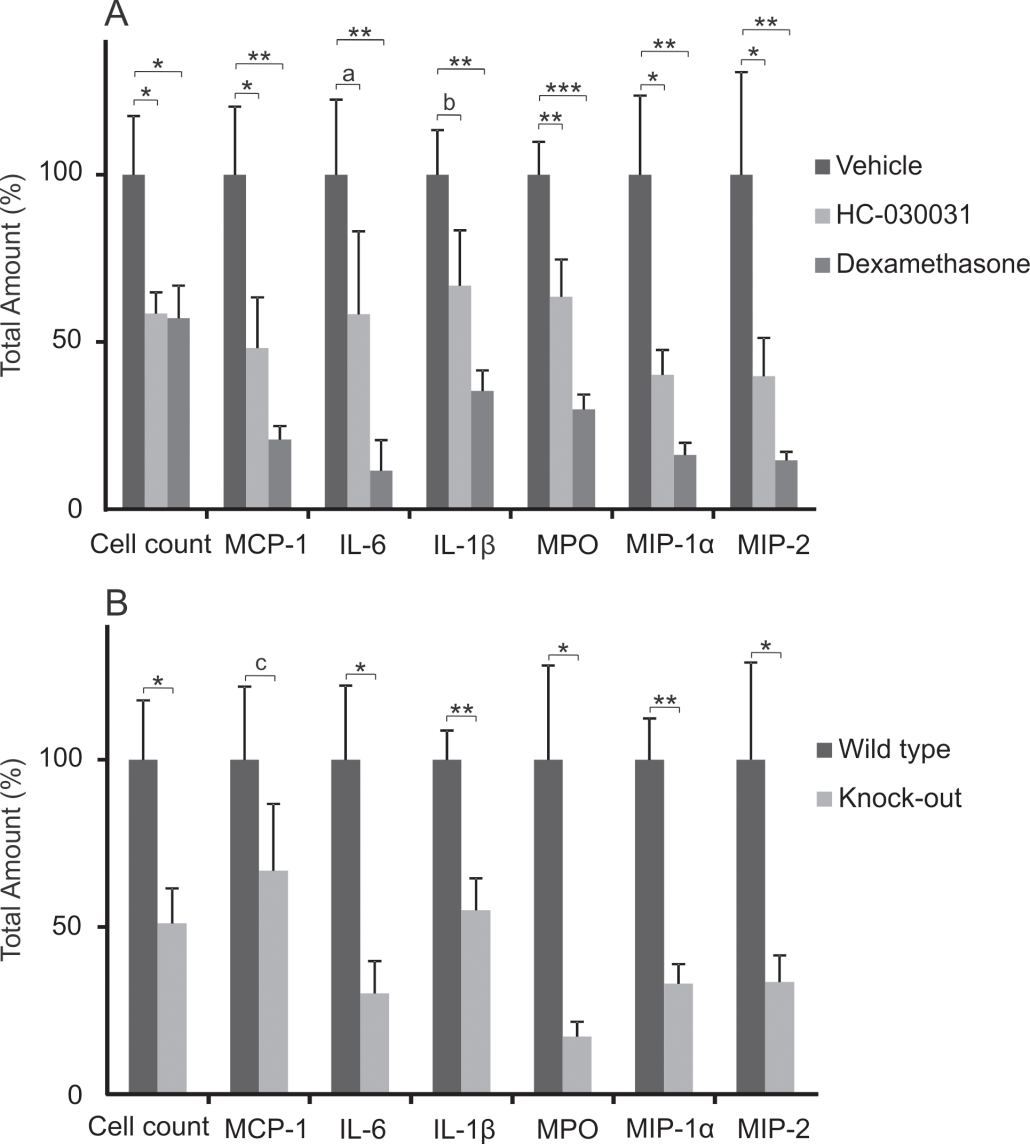
Effect of TRPA1 Activation in MSU Crystal-Induced Air-Pouch Test. Treatment with TRPA1 antagonist HC-030031 (300 mg/kg) or dexamethasone (2 mg/kg) inhibited monosodium urate (MSU) crystal-induced production of proinflammatory cytokines MCP-1, IL-6, IL-1β, MPO, MIP-1α and MIP-2 and accumulation of cells in the synovial joint resembling subcutaneous air-pouch model in the mouse (A). Accordingly, genetic depletion of TRPA1 led to an attenuated inflammatory response when compared to corresponding wild type mice (B). The studied drugs were given orally 2h prior to 3 mg of MSU crystals diluted in 1 ml of endotoxin free phosphate buffered saline were injected into the air-pouch. The exudate was harvested 6h after the MSU crystal injection and cells were counted using hemocytometer and the cytokines were analysed using ELISA. Results are displayed as total amount of cells or cytokines per air-pouch. The mean amount of the vehicle treated mice was set as 100% and the other values are related to that. The results are expressed as mean + SEM, n = 7–8, a indicates p = 0.129, b indicates p = 0.067, c indicates p = 0.148, * = p<0.05, ** = p<0.01, *** = p<0.001.

Monosodium urate (MSU) crystals induced an acute inflammatory response when injected into the subcutaneous air-pouch of studied mice. The response was characterized by accumulation of cells and inflammatory cytokines monocyte chemotactic protein-1 (MCP-1), interleukin-6 (IL-6), interleukin-1β (IL-1β), myeloperoxidase (MPO), macrophage inflammatory protein-1α (MIP-1α) and macrophage inflammatory protein-2 (MIP-2) (S1 Table). The acute inflammatory response induced by injection of MSU crystals into subcutaneous air-pouch was remarkably inhibited by treatment with the selective TRPA1 antagonist HC-030031 or with dexamethasone which was used as a control compound with known anti-inflammatory activity. Interestingly, HC-030031 treatment significantly inhibited the total accumulation of cells (42% inhibition, p<0.05) and proinflammatory cytokines MCP-1, MPO, MIP-1α, MIP-2 (37–60% inhibition, p<0.05 or p<0.01) and a similar trend was seen in IL-6 (42% inhibition, p = 0.129) and IL-1β (33% inhibiton, p = 0.067). As expected, the total accumulation of cells and proinflammatory mediators was significantly attenuated also by dexamethasone. Comparable results to those seen in pharmacological inhibition of TRPA1 were also observed when responses in TRPA1 KO and WT mice were compared. In KO mice the total amounts of IL-6, IL-1β, MPO, MIP-1α, and MIP-2 were attenuated in a statistically significant manner (45–83% reduction, p<0.05 or p<0.01) as compared to the corresponding WT mice and a non-significant reduction in MCP-1 production (33% reduction, p = 0.148) was observed. (Fig. 1A and 1B)

**Fig 2:**
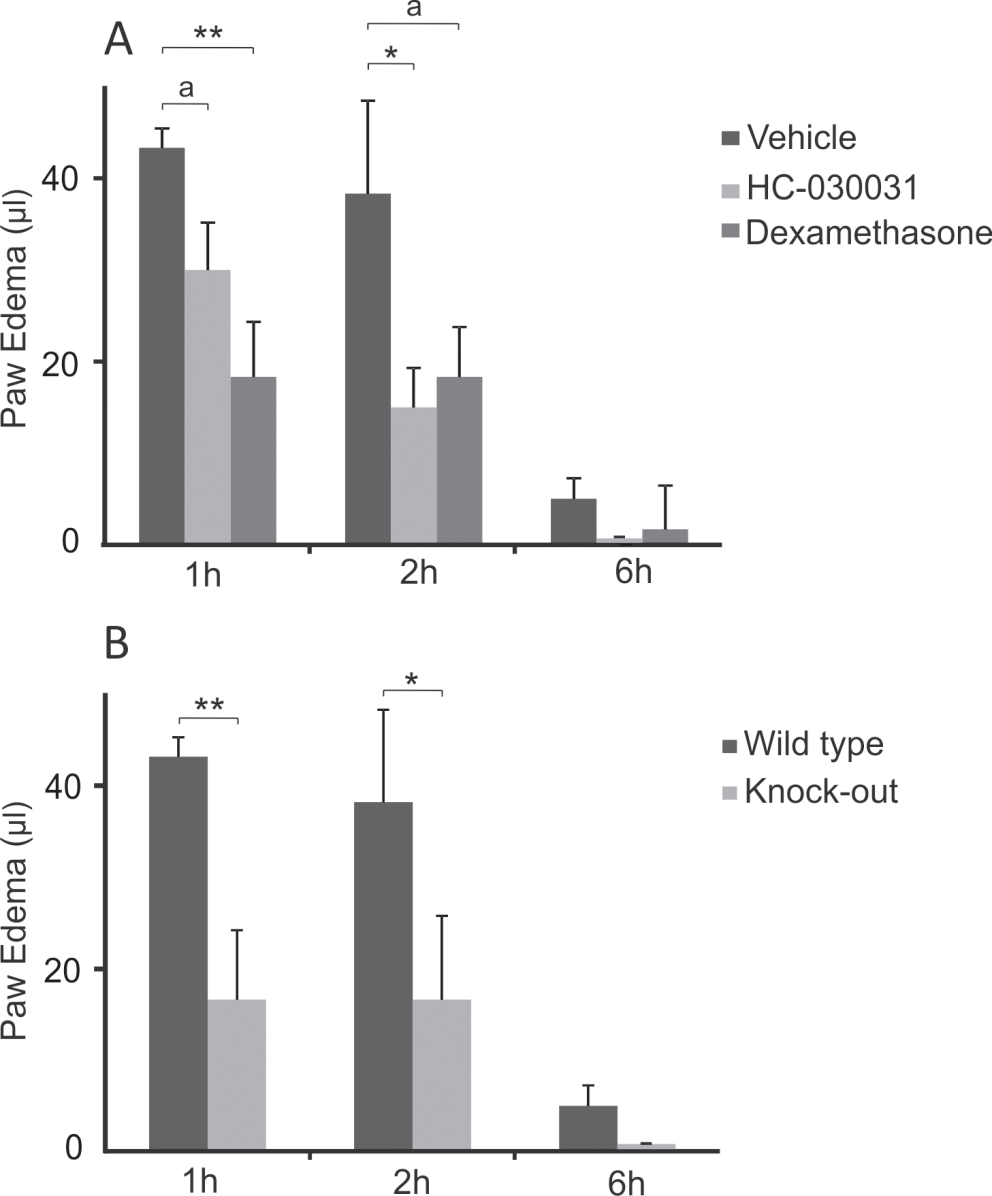
Effect of TRPA1 Activation in MSU Crystal-Induced Inflammatory Paw Edema. Treatment with TRPA1 antagonist HC-030031 (300 mg/kg) or dexamethasone (2 mg/kg) inhibited mouse paw edema formation induced by an injection of monosodium urate (MSU) crystals (A). In non-treated mice, TRPA1 deficiency caused an alleviated edema formation compared to the corresponding wild type mice (B). The studied drugs were given orally 2h prior to the initiation of the experiment by injecting 0.5 mg of MSU crystals in 40 μl of endotoxin free phosphate buffered saline into the mouse hind paw. The paw volume was measured with a plethysmometer before and up to 6h after MSU crystal injection. The contralateral control paw injected with the vehicle developed no measurable edema. Paw edema is expressed as the volume change as compared to the pre-treatment value and the results are displayed as mean + SEM, n = 5–6, a indicates p = 0.057, * = p<0.05, ** = p<0.01.

Intraplantar injection of MSU crystals induced an acute inflammatory edema (Fig. 2). The MSU crystal-induced paw edema was clearly attenuated by the treatment with the selective TRPA1 antagonist HC-030031 (61% inhibition at 2h, p<0.05) or the positive control compound dexamethasone. Accordingly, TRPA1 KO mice developed significantly less severe edema than corresponding WT mice (57% reduction at 2h, p<0.05). (Fig. 2A and 2B)

**Fig 3:**
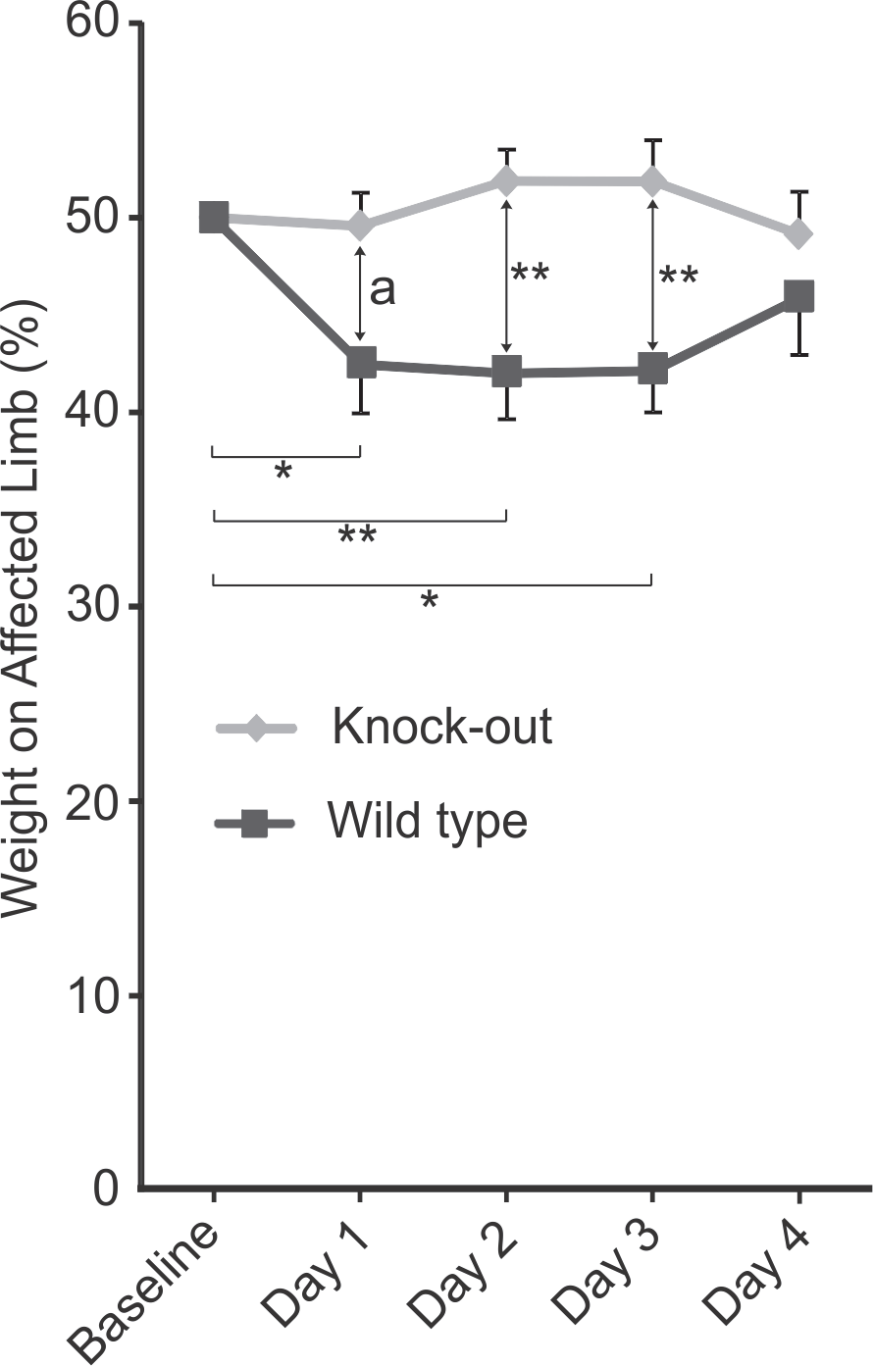
Effect of TRPA1 Activation in MSU Crystal-Induced Spontaneous Weight-Bearing Test. Spontaneous weight-bearing on hind limb knee joint injected with monosodium urate (MSU) crystals indicative of joint pain was altered in wild type but not in TRPA1 deficient mice. The difference between the groups was also significant. An injection of 0.5mg of MSU crystals diluted in 40 μl of endotoxin free phosphate buffered saline into the knee joint was performed and the mice were measured for spontaneous distribution of weight between hind limbs with an incapacitance meter for four subsequent days and referred to the basal level. The contralateral knee was injected with the vehicle only and the measurer was blinded for the affected limb. Results are displayed as the percentage of weight bore by the affected limb and given as mean + SEM, n = 7–10, * = p<0.05, ** = p<0.01.

In the weight-bearing test indicating joint pain, WT mice developed a clear reduction in spontaneous weight-bearing on the affected limb when measured 1, 2 and 3 days after an intra-articular injection of MSU crystals and the weight distribution was normalized at the 4^th^ day. Interestingly, injection of MSU crystals did not induce any weight distribution change in TRPA1 deficient mice. (Fig. 3)

**Fig 4:**
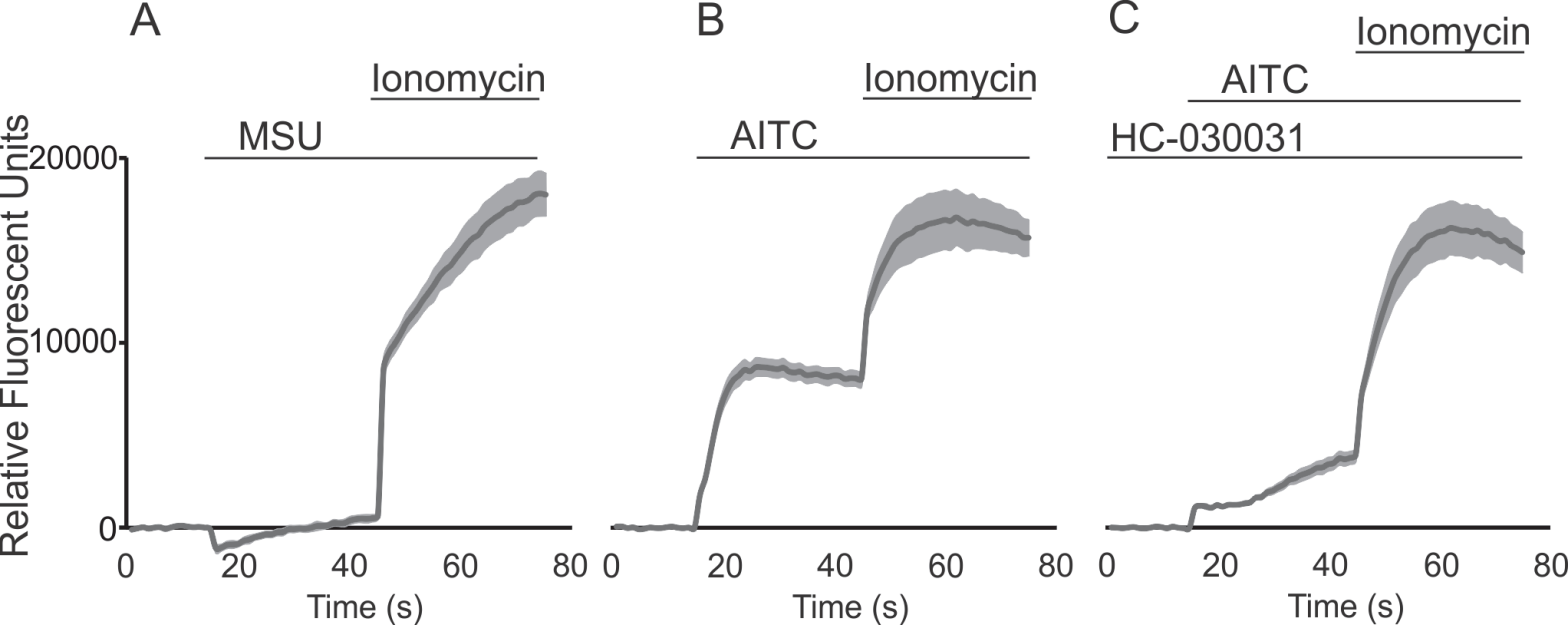
Effect of MSU Crystals in TRPA1-Mediated Ca^2+^-Influx in HEK293 Cells. Monosodium urate (MSU) crystals did not evoke TRPA1-mediated Ca^2+^-influx (A) whereas the known TRPA1 agonist allyl isothiocyanate (AITC) induced a robust Ca^2+^-influx (B) which was inhibited by pre-treatment with the selective TRPA1 antagonist HC-030031 (C). HEK293 cells were transfected with plasmids encoding TRPA1 and loaded with Fluo-3 AM as described in the Methods. The intracellular Ca^2+^ concentration was assessed by Victor3 multilabel counter at excitation/emission wavelengths of 485/535 nm at 1/s frequency. The cells were first pre-incubated with 100 μM HC-030031 (C) or the vehicle (A-B) for 30 min at +37 C°. In the experiments (A-C) basal fluorescence was first measured for 15s and thereafter 1 mg/ml of MSU crystals (A) or 50 μM AITC (B-C) was added and the measurement was continued for 30s after which 1 μM of the control ionophore compound ionomycin was introduced to the cells. The seen transient minor fall in the relative fluorescent units after the application of MSU (A) is regarded as an artifact as a similar phenomenon is often seen following an application of inert compounds or solvents into the wells. The results are normalized against background and expressed as mean (drak gray line) ± SEM (light gray shadowing), n = 6.

Direct activation of TRPA1 by MSU crystals was excluded by studying Ca^2+^-influx in TRPA1-transfected HEK293 cells. MSU crystals did not evoke Ca^2+^-influx to the TRPA1-transfected cells indicating that MSU crystals do not function as a direct TRPA1 agonist. In contrast, introduction of AITC to the cells induced a strict increase in the intracellular Ca^2+^ concentration which was inhibited by the TRPA1 antagonist HC-030031. (Fig. 4A-C)

## Discussion

The present results show that TRPA1 has a significant role in the development of MSU crystal-induced joint pain, inflammatory mediator production and edema formation as evidenced by the effects of pharmacological blockade and/or genetic depletion of TRPA1 in murine models.

In the present study, three models of MSU crystal-induced response were investigated. The air-pouch inflammation model is a widely used model to mimic synovial inflammation enabling the harvest of inflammatory exudate which is extremely difficult to sample reliably from the very small mouse joints. The results show that TRPA1 mediates the MSU crystal-induced accumulation of inflammatory cells (mainly neutrophils), and inflammatory cytokines into the air-pouch. Secondly, we used paw inflammation model to measure inflammatory edema related to soft-tissue inflammation, and found that TRPA1 mediates also the MSU crystal-induced inflammatory edema formation which is linked to vascular leakage. Thirdly, we used weight-bearing test to estimate joint pain as nociceptive responses were not possible measurements in the two previous models as their license allows to use only anesthetized mice in those experiments. According to the results of the weight-bearing test, TRPA1 mediates also the MSU crystal-induced joint pain. Intriguingly, Ferreira and Geppetti with their co-workers published very recently two articles reporting that activation of TRPA1 contributes to MSU crystal-induced responses when MSU crystals were injected in the rodent paw [17] or ankle joint [18]. They found that TRPA1 antagonist was able to inhibit MSU crystal-induced acute edema, nociception, production of IL-1β and myeloperoxidase and accumulation of neutrophils. They also discovered that genetic depletion of TRPA1 reduced the acute nociceptive and edematogenic effects of TRPA1. Those findings highly support the results of the current study. Further, the present results extend the previous knowledge by providing information on air-pouch model and on a broad range of proinflammatory cytokines, and by extending the studied time span of pain-like behavior resulting from MSU crystal injection as demonstrated in the spontaneous weight bearing test. These three studies beautifully complement and strengthen each other and highlight the previously unknown role of TRPA1 in the development of MSU crystal-induced inflammation and pain applicable for the pathogenesis and drug development for gout flare.

TRPA1 activation has proved to mediate nociception and neurogenic and inflammatory pain in various experimental models [5,6,13]. Also, TRPA1 hyperfunction due to genetic mutation was recently reported to be associated with a severe familiar episodic pain syndrome proving the significant role of TRPA1 in human pain [31]. The present results together with those reported by Ferreira’s and Geppetti’s groups [17,18] show that TRPA1 mediates joint pain in a mouse model of MSU crystal-induced arthritis adding MSU crystals into the list of painful compounds mediating their effects through indirect activation of TRPA1 channels. Further, the results strongly suggest TRPA1 as a mediator and drug target to treat painful gout flares in man.

Several irritating exogenous compounds known to cause inflammatory edema in the skin, gut or respiratory track have appeared as direct activators of TRPA1 [5,6]. Interestingly, in addition to sensing exogenous irritating/proinflammatory compounds, TRPA1 has also been reported to be an endogenous mechanism mediating inflammatory edema highlighted by the fact that many compounds not directly interacting with TRPA1 cause an inflammatory reaction yet dependent on the activation of TRPA1 [5,6,19]. The TRPA1 triggered inflammatory edema – which is usually associated with hyperalgesia – has been proposed to be connected to several classical inflammatory mechanisms such as mast cell degranulation, neutrophil migration, release of histamine, serotonin and adrenalin and production of prostaglandins [19,32]. Furthermore, activation of TRPA1 has been reported to enhance the expression of inflammatory genes including IL-1β and the prostaglandin producing enzyme cyclooxygenase-2 in certain experimental models [19,33,34]. Accordingly, we found in the present study that pharmacological inhibition and genetic depletion of TRPA1 reduced the production of several MSU crystal-induced chemokines and inflammatory cytokines as measured in the air-pouch model. Even though mechanisms related to TRPA1-triggered changes in gene expression are not fully understood, TRPA1-induced increase in intracellular Ca^2+^ concentration may directly mediate the effect as changes in intracellular Ca^2+^ levels are known to regulate the transcription of some inflammatory genes [35–37]. Against this background, our present findings, in which inhibition or depletion of TRPA1 significantly reduced edema formation and accumulation of proinflammatory cytokines and inflammatory cells in response to MSU crystals in synovial joint resembling subcutaneous air-pouch and in subcutaneous soft-tissue are reasonable and together with the results described recently [17,18] support the role of TRPA1 as a mediator of inflammatory responses in acute gouty arthritis.

Presentation of MSU crystals to the inflammatory cells triggers several inflammatory mechanisms including ROS production and activation of PI3K and NF-κB pathways and NALP3 inflammasome. Activation of NALP3 leads to the release of powerful proinflammatory cytokine IL-1β [2,3]. In animal models, elimination of IL-1β has been reported to cause a clear but not a total inhibition of inflammation and pain suggesting the presence of additional mechanisms mediating the responses [4]. The present study proposes TRPA1 as such a mechanism. However, in the intracellular Ca^2+^ measurements MSU crystals were found not to directly activate TRPA1. An interesting hypothetical link between TRPA1 and MSU crystals lies, however, in the fact that MSU crystals induce oxidative stress and ROS production [2,3]. Reactive oxygen species are involved in the activation of NALP3 inflammasome and other cellular responses to MSU crystals [2,3]. Notably, multiple products of oxidative stress e.g. hydrogen peroxide and 4-hydroxynonenal are also known to activate TRPA1 which could amplify the inflammatory response either by NALP3 dependent or independent mechanism following exposure to MSU crystals [6,15,17,18].

TRPA1 mediated inflammation is frequently explained by the neuronal expression of TRPA1 and the release of proinflammatory neuropeptides following its activation [6,13]. Calcitonin gene related peptide and substance P are best known of those neuropeptides which mediate the neurogenic inflammation triggered by activation of neuronal TRPA1. Curiously, the release of substance P from the surrounding neurons has been previously proposed in an experimental MSU crystal-induced arthritis [38] promoting neurogenic inflammation also as a potential mechanism involved in the formation of gouty arthritis. The role of neuronal mechanism in the development or MSU crystal-induced inflammation is supported by the finding that neonatal dysfunctionalization of peptidergic nerve fibers by capsaicin attenuates the pain induced by MSU crystals [24]. It is of note that the neurons which express the capsaicin sensor TRPV1 often co-express TRPA1 [8]. However, the non-neuronal TRPA1 expression and function has been clearly established in many cell types such as keratinocytes, synoviocytes and endothelium [39] giving rise to a possibility that MSU crystal-induced inflammation is also linked to the non-neuronal TRPA1 activation. Descriptively, activation of TRPA1 in vascular endothelium in addition to vascular sensory neurons is capable of causing edema through vasodilatation and increased vascular permeability [40]. Also, increase in intracellular Ca^2+^ concentration via activated TRPA1 ion channel may regulate the expression and release of inflammatory factors in both inflammatory and resident tissue cells as discussed above [35–37]. In our study we provide data originating from animal models focusing on tissue and organism level results of TRPA1 activation following the application of MSU crystals indicating that the responses are functional *in vivo* and not restricted to experimental *in vitro* conditions. Based on these results it is not, however, possible to define the cell types responsible for TRPA1 mediated responses in MSU crystal-induced inflammation yet it is likely that TRPA1 convoyed pro-inflammatory responses are due to the interaction between different neuronal and non-neuronal cell types.

In addition to the mechanisms described above, a more direct interaction between TRPA1 and IL-1β in MSU crystal-induced inflammation is possible, as TRPA1 activation has been reported to enhance IL-1β production in keratinocytes [33], and a similar effect was found in the present study in the air-pouch model. On the other hand, IL-1β has been found to enhance TRPA1 expression in human synovial cells especially in hypoxic conditions [41]. Although several potential mechanisms do exist, the exact means how TRPA1 exacerbates the MSU crystal-induced gouty inflammation cannot be conclusively defined by the present results but warrants further studies.

Currently, treatment of gout flare includes systemic or local corticosteroids, non-steroidal anti-inflammatory drugs and colchicine [1] but there is a real need for novel treatment modalities, especially in resistant cases. The present results propose TRPA1 as an attractive novel anti-inflammatory and analgesic drug target to treat acute gouty arthritis.

## Conclusions

TRPA1 was found to mediate MSU crystal-induced inflammation and pain in three different experimental models supporting the role of TRPA1 as a potential mediator and a drug target in gout flare.

## Supporting Information

S1 Table Monosodium urate (MSU) crystals induced accumulation of inflammatory cytokines and cells into synovial joint mimicking subcutaneous air-pouch in the mouse. The amounts of accumulated cells and of proinflammatory cytokines monocyte chemotactic protein-1 (MCP-1), interleukin-6 (IL-6), interleukin-1β (IL-1β), myeloperoxidase (MPO), macrophage inflammatory protein-1α (MIP-1α) and macrophage inflammatory protein-2 (MIP-2) measured in the synovial joint resembling subcutaneous air-pouch inflammation model in the mouse. The studied mice were injected into the air-pouch with 3 mg of monosodium urate (MSU) crystals in 1 ml of endotoxin free phosphate buffered saline (PBS) or with 1 ml of PBS only. The exudate was harvested 6h after the injection and cells were counted using hemocytometer and the cytokines were analysed using ELISA. Results are displayed as total amount of cells or cytokines per air-pouch. The results are expressed as mean ± SEM, n = 5–8, * = p<0.05, ** = p<0.01, *** = p<0.001.
(PDF)
