# Supplementary material for: Urate Crystal Induced Inflammation and Joint Pain Are Reduced in Transient Receptor Potential Ankyrin 1 Deficient Mice – Potential Role for Transient Receptor Potential Ankyrin 1 in Gout
Source: PLoS One. 2015 Feb 6;10(2):e0117770. doi: 10.1371/journal.pone.0117770 (PMC4319920; doi:10.1371/journal.pone.0117770)
Supplement: S1 Table — The amounts of accumulated cells and of proinflammatory cytokines monocyte chemotactic protein-1 (MCP-1), interleukin-6 (IL-6), interleukin-1β (IL-1β), myeloperoxidase (MPO), macrophage inflammatory protein-1α (MIP-1α) and macrophage inflammatory protein-2 (MIP-2) measured in the synovial joint resembling subcutaneous air-pouch inflammation model in the mouse. The studied mice were injected into the air-pouch with 3 mg of monosodium urate (MSU) crystals in 1 ml of endotoxin free phosphate buffered saline (PBS) or with 1 ml of PBS only. The exudate was harvested 6h after the injection and cells were counted using hemocytometer and the cytokines were analysed using ELISA. Results are displayed as total amount of cells or cytokines per air-pouch. The results are expressed as mean ± SEM, n = 5–8, * = p<0.05, ** = p<0.01, *** = p<0.001. (PDF) [file pone.0117770.s001.pdf]

Supportive information Table S1

Monosodium urate (MSU) crystals induced accumulation of inflammatory cytokines and cells into synovial joint mimicking subcutaneous air-pouch in the mouse

Group	Cell Count ($1x10^6$)	MCP-1 (pg)	IL-6 (pg)	IL-1β (pg)	MPO (ng)	MIP-1α (pg)	MIP-2 (pg)
MSU	1.205 ± 0.211**	1946.6 ± 396.0*	184.1 ± 41.2**	37.6 ± 5.0*	32.63 ± 3.20***	142.2 ± 33.6**	256.1 ± 78.5*
PBS	0.118 ± 0.021	411.6 ± 105.8	5.9 ± 1.8	17.1 ± 0.8	6.72 ± 0.75	6.4 ± 1.4	<7.8 ± 0

The amounts of accumulated cells and of proinflammatory cytokines monocyte chemotactic protein-1 (MCP-1), interleukin-6 (IL-6), interleukin-1β (IL-1β), myeloperoxidase (MPO), macrophage inflammatory protein-1α (MIP-1α) and macrophage inflammatory protein-2 (MIP-2) measured in the synovial joint resembling subcutaneous air-pouch inflammation model in the mouse. The studied mice were injected into the air-pouch with 3 mg of monosodium urate (MSU) crystals in 1 ml of endotoxin free phosphate buffered saline (PBS) or with 1 ml of PBS only. The exudate was harvested 6h after the injection and cells were counted using hemocytometer and the cytokines were analysed using ELISA. Results are displayed as total amount of cells or cytokines per air-pouch. The results are expressed as mean ± SEM, n=5-8, *=p<0.05, **=p<0.01, ***=p<0.001
